# Quality care and safety know no borders

**DOI:** 10.2349/biij.3.3.e34

**Published:** 2007-07-01

**Authors:** JP Borgstede, PA Wilcox

**Affiliations:** Department of Quality and Safety, American College of Radiology, Reston, Virginia, United States

**Keywords:** Quality, safety, radiology

## Abstract

The public, governmental agencies, and payers expect medical professional organisations to develop practice guidelines and technical standards. The American College of Radiology proactively addresses these topics as well as other quality and safety interests including appropriateness criteria and accreditation. The College is also actively involved in development of a national radiology data base to collect data regarding quality and safety metrics in multiple areas. In addition, the College has developed RADPEER™, a simple, cost-effective process that allows peer review to be performed during the routine interpretation of current images. This paper discusses the efforts of the ACR in all of these areas.

## INTRODUCTION

Eighty-three years ago, a group of radiologists gathered around a table in a San Francisco, California, hotel and laid the groundwork for the American College of Radiology (ACR), an organisation committed to the ideals of quality, appropriate, and safe radiologic care. Today, that commitment remains stronger than ever and is reflected in the College’s motto – “Quality is Our Image.” That group of radiologists, motivated by a desire to instil ethics in the new developing medical field of radiology and excited about the technological opportunities to improve patient care, recognised the need to insure quality in this evolving specialty. In the years since they followed their shared vision of professional excellence, our profession has seen some of the most exciting and rewarding technical advancements known to medicine. A medical specialty that commenced with a single X-ray of the hand of Wilhelm Roentgen’s wife now offers an array of imaging tools that allow us to diagnose and treat patients with an exactness that was inconceivable even a decade ago. Today, being a radiologist means more than interpreting an image. A radiologist is a consultant with a capability of integrating medical physics, pathophysiology, and medicine.

As our knowledge and methods of imaging have changed through the years, so has our ability to share information across international borders thereby strengthening our professional bond with our colleagues abroad. We share a common commitment and obligation to the diagnosis and treatment of diseases that do not recognise differences in politics, differences in creeds, or differences in philosophies. As a global radiologic community, we are witnesses to a new technological era with new imaging and treatment tools ranging from molecular imaging to picture archival systems (PACS) with which to transmit our images around the world. The American College of Radiology is proud to be a leader in setting the radiologic standard of quality and safety in so many facets of our profession and the College values the opportunity today’s electronic world offers for the open and beneficial exchange of knowledge and information. It is the responsibility of each member of the radiologic community, and the organisations to which they belong, to participate in an open exchange regarding tools for improving quality and safety in imaging.

## ACR ACCREDITATION PROGRAMS – THE HALLMARK OF QUALITY

Since 1963, ACR accreditation has been the recognised sign of quality for radiologic facilities throughout the United States. The College’s history of developing and administering accreditation programs to assess a facility’s level of quality originated with the Diagnostic Practice Accreditation Program in 1963. Twenty years ago, the ACR took its next important accreditation step with a focus on improving the quality of mammography through the Mammography Accreditation Program. With this step, the ACR accepted responsibility for improving breast imaging at a time when the public, payers, and governmental agencies were questioning mammographic quality [1].

Fuelled by the success of that program, and driven by the desire to set quality standards in other areas of imaging, the ACR developed eight other, modality-specific accreditation programs to ensure that patients receive high quality imaging. In recent years, these programs have taken on an even greater relevance as government and third-party payers demand pay for performance (P4P) metrics and evidence that more imaging equates to better patient care. The ACR’s array of accreditation programs will continue to adapt to meet the needs and demands of patients, imaging specialists, and payers.

## PRACTICE GUIDELINES AND TECHNICAL STANDARDS: A MAP TO QUALITY CARE

Originating in the 1930s, the ACR’s Practice Guidelines and Technical Standards define the principles, technical parameters, and acceptable methods in diagnostic radiology, radiation oncology, and medical physics to diagnose and treat typical patients in typical circumstances to produce desired health care outcomes[2]. The practice guidelines and technical standards are reviewed every five years to ensure relevance to current radiologic practices. Those guidelines or standards with substantive changes undergo revision and are subject to a fresh review process. New or revised guidelines or standards must be approved by the ACR Council to be accepted as official ACR policy. This approval process further ensures their relevance. Recent examples of guideline revisions include the ACR guidelines on communication, MRA, and CTA. Table 1 and Table 2 summarise some of the new guidelines proposed for 2007.

**Table 1:** ACR Practice Guidelines and Technical Standards for 2007.

ACR Practice Guideline for Performing FDG-PET/CT in Oncology
ACR Technical Standard for Electronic Practice of Medical Imaging
ACR Practice Guideline for the Performance of Single Photon Emission CT (SPECT) Brain Perfusion and Brain Death Studies

**Table 2:** Collaborative Practice Guidelines for 2007.

Practice Guideline for the Performance of CT Perfusion in Neuroradiologic Imaging **(ASNR)**
Practice Guideline for the Performance of Functional Magnetic Resonance Imaging of the Brain **(fMRI) (ASNR)**
Practice Guideline for the Performance of Intracranial Magnetic Resonance Bolus Perfusion Imaging **(ASNR)**
Practice Guideline for the Performance of MRI of the Wrist **(SSR)**
ACR Practice Guideline for the Performance of Vascular Ultrasound for Postoperative Assessment of Dialysis Access **(AIUM)**
ACR Practice Guideline for the Performance of an Ultrasound Examination of the Neonatal Spine **(AIUM)**
Practice Guideline for Determinants of Image Quality in Digital Mammography **(AAPM, RSNA, SIIM)**
Practice Guideline for Digital Radiography **(AAPM, SIIM)**
Practice Guideline for the Performance of Physiologic Evaluation of Extremity Arteries **(AIUM, SIR)**
ACR Practice Guideline for the Performance of Transcranial Doppler US **(AIUM)**
ACR Practice Guideline for the Performance of the Musculoskeletal Ultrasound Examination **(AIUM)**

Currently, the ACR faces the challenge of transitioning our practice guidelines into performance measures and quantifiable quality indicators to meet governmental, payer, and public expectations. It is important that radiologists and the ACR lead rather than follow in this effort lest the public, private payers, and governmental agencies direct these efforts in pathways less optimal for patient care. To this end the ACR has formed a Committee on Metrics that is developing meaningful process, structure and outcomes measures for general radiology. Additionally, we have initiated the National Registry for Diagnostic Radiology. This data warehouse will incorporate multiple registries including the National Oncologic PET Registry (NOPR), the National Carotid Stent Registry (NCR), the CT Colongraphy Registry (CTCR), the Cardiac CT Registry (CTCR), the National Mammography Data Registry (NMD), the General Radiology Improvement Database (GRID), and the Dose Index Registry. Through the collection and analysis of data from these registries we will be able to set benchmarks for quality radiologic care and provide guidance to radiologists for continuous quality improvement.

## THE APPROPRIATENESS OF RADIOLOGIC CARE

In 1993, in response to requests from ACR members and referring physicians, as well as pressures from third-party payers, the ACR’s leadership approved the development of the ACR Appropriateness Criteria^®^ which are a compilation of evidence-based recommendations designed to assist referring physicians and other providers in their choices of the most appropriate imaging examination or treatment for a given clinical condition [3]. The Appropriateness Criteria^®^ are designed by expert panels representing the fields of diagnostic imaging, interventional radiology, and radiation oncology and now cover more than 170 topics with more than 900 variants, addressing most common disease entities where there is a high volume of imaging, variations in practice, or high-risk procedures. Imaging modalities are ranked on a 1-9 scale with 1 being the least appropriate modality and 9 being the most appropriate modality for a given clinical condition based on a metanalysis of the scientific literature. An example of the use of the Appropriateness Criteria^®^ would be in evaluation of low back pain and the appropriateness of imaging and the choice of imaging modality (Table 3).

**Table 3:** American College of Radiology ACR Appropriateness Criteria^®^. Clinical Condition: Low Back Pain Variant 1: Uncomplicated. No red flags. (Red flags defined in text.)

**Radiologic Procedure**	**Appropriateness Rating**	**Comments**
X-ray, lumbar spine	2	
NM, bone scan	2	
CT, lumbar spine, without contrast	2	
X-ray, myelography, lumbar spine	2	Usually done in conjunction with CT.
CT myelography, lumbar spine	2	Usually accompanied by plain film myelogram.
MRI, lumbar spine, without contrast	2	
MRI, lumbar spine, without and with contrast	2	

Appropriateness Criteria^®^ Scale 1 2 3 4 5 6 7 8 9 1=Least appropriate 9=Most appropriate

The Appropriateness Criteria^®^ has received acceptance from outside the radiologic community. Currently, several third-party payers have expressed an interest in applying the Appropriateness Criteria^®^ to ensure quality imaging and contain rising imaging-related costs. In addition, other vendors and payers are seeking to incorporate the Criteria into their products, offering additional opportunities for the ACR to ensure continued delivery of quality imaging care. To allow for wider and easier access to the Appropriateness Criteria^®^, the ACR introduced a downloadable PDA version and posts updated material on its Web site. Each of the Criteria’s topics is reviewed annually and, based on current medical literature and key practice trends, either a complete or an administrative review is performed. In summer of 2007 the newest version of the Appropriateness Criteria^®^ will be launched. It will include an improved search capability, ICD-9 codes, CPT codes and guidance on dose levels for various imaging examinations. The ACR is actively working with primary care physicians such as the American Academy of Family Practice and the American College of Physicians to disseminate the Appropriateness Criteria^®^ to the referring physician community. We believe the Appropriateness Criteria^®^ can have a major impact on reducing the spiralling costs from inappropriate medical imaging while helping to ensure the most effective use of imaging and imaging guided therapy [4].

## RADIOLOGISTS’ PERFORMANCE IN PRACTICE

The College has also developed a peer review program, RADPEER™, to assess the performance of radiologists in practice. Again, as with facilities, the public, payers, the American Board of Radiology, and the government require documentation, through peer review, that radiologists are performing daily with skill and safety. The RADPEER™ program was designed by the ACR to document radiologists’ performance and to identify areas for improvement [5]. This program is based on the premise that when a radiologist interprets an imaging study and compares his or her current impression with the interpretation of the previous examination, a peer review event has occurred. RADPEER™ simply applies a 1 through 4 scoring system to the levels of agreement or disagreement between the current and previous interpretations. This data is collected by the ACR and allows individual radiologists to confidentially compare their performance to those of their peers and to focus their education toward areas optimal for improvement.

## TARGETED QUALITY PROGRAMS

The programs discussed above are all programs spanning the gamut of radiology. The College has also developed other targeted programs to meet unique requirements and issues. Below are examples of such programs.

### BI-RADS®

The Breast Imaging Reporting and Data System (BI-RADS®) Atlas, is a comprehensive lexicon and reporting system developed by the College as a reference for education, consistent terminology and uniform reporting of breast imaging including mammography, ultrasound and MRI [6]. The BI-RADS® system provides for clear communication of findings to referring physicians and other radiologists to help guide patient care. BI-RADS® is recognised and used by breast imagers world-wide.

### ACR Guidance Document for Magnetic Resonance Safe Practices

In 2002, in response to concerns regarding MR safety and adverse incidents involving patients, equipment, and personnel, the College convened a Blue Ribbon Panel on MR Safety. The Panel was charged with reviewing MR safety practices and guidelines and issuing new ones as appropriate for MR examinations and practices. This Panel published its original document in 2002 and has twice revised and updated the document-most recently in 2007 [7, 8]. This document provides guidelines specific to MR sites, patient screening, and practices as they relate to MR safety. The panel’s recommendations have been published in the American Journal of Roentgenology.

### ACR White Paper on Radiation Dose in Medicine

As a result of the increased utilisation of imaging using ionising radiation, particularly CT, the ACR convened a Blue Ribbon Panel on Radiation Dose in Medicine to address the issue of increased dose to patients and the potential for increased incidence of cancer. The white paper includes recommendations for educating the public, referring providers and the radiology community as well ways to prevent inappropriate imaging while still optimising the quality of studies at the lowest possible dose. This paper was published in the May 2007 issue of the Journal of the American College of Radiology [9].

## TELERADIOLOGY AND QUALITY

The ACR originally introduced its Technical Standard for Teleradiology in 1994. This document has most recently been updated in 2006 [10]. The technical standard defines the goals, qualifications of personnel, equipment guidelines, licensing, credentialing, liability, communication, quality control, and quality improvement for teleradiology.

PACS combined with the ability to transmit images electronically have proven a challenge to the public and to radiologists. In response to quality concerns in this area, the ACR Task Force on International Teleradiology was convened in the summer of 2003 with the explicit goal of studying legal, regulatory, reimbursement, quality assurance, and other key issues associated with the emerging practice. The resulting white paper on teleradiology, published in 2004, recommends that overseas’ radiologists to adhere to the same educational and professional standards for interpreting radiologic images as their American counterparts [11]. The paper also recommends radiologists involved in teleradiology be licensed in the sending and receiving states, participate in the sending site’s quality control programs, and prohibits “ghosting” of reports. Ghosting is a terminology referring to the attribution of an imaging report to a physician other than the actual interpreting physician.

## THE ACR’S RESPONSIBILITY TO INTERNATIONAL STAGE

The ACR and its members recognise that we do not practice health care in a vacuum and that our responsibility to quality imaging and our collective patients extends far beyond our own borders. Our commitment to quality patient care requires us to look beyond political and cultural differences to address humanity’s urgent medical needs and fulfil our obligation to support the highest quality care possible. In recent years, the College and its leadership have taken a prominent role on the worldwide radiologic stage in order to forge new and productive relationships with radiologists abroad as we focus on our common goal of quality medical imaging. These efforts include the development of the ACR Committee on International Service. A part of this committee’s goals are to promote quality imaging abroad through international service and contributions by ACR members. International efforts in quality also include the distribution of ACR Commission on Quality and Safety materials to practices in developing countries. The ACR has supported education for radiologists and government agencies on mammography accreditation in Turkey, the Philippines and later this year in Kenya. Additionally, both BI-RADS^®^ and the ACR Mammography Quality Control Manual have been translated into many languages and are used in countries around the world.

Furthermore, the ACR, in conjunction with industry, has recently initiated a program bringing Iraqi radiologists to US sites for updated training to improve the quality of imaging as they return to their country.

The ACR is also a board member of the International Radiology Quality Network (IRQN) that was established by Professor Lawrence Lau of Australia. This group is working collaboratively to develop guidance for teleradiology that all participating countries can agree to. In addition, in 2007 the ACR has volunteered to use its experience with registry development to begin an international pilot project to collect practice improvement data. The initial project will relate to report turn around time as compared to the volume of exams, FTE radiologists and FTE technologists. If it is successful the international registry will be expanded to include other measures similar to those that are being developed by the Committee on Metrics for GRID.

## CONCLUSION

Quality in imaging extends far beyond what a few physicians, calling themselves by the new term “radiologist” could ever have envisioned 83 years ago at that meeting in San Francisco. From a few individual rudimentary quality programs in the past to an entire commission devoted to quality and safety including 40 committees with over 1000 dedicated volunteers and a staff of over 50 individuals including radiologic technologists, nurses, and lawyers the American College of Radiology maintains a steadfast commitment to a statement that is more than our logo Quality ***IS*** Our Image!
